# Poor sleep quality remains a major challenge among tertiary education students in Ghana: A cross-sectional study in a Ghanaian University

**DOI:** 10.1016/j.ibneur.2024.12.014

**Published:** 2024-12-31

**Authors:** David Larbi Simpong, Ansumana Bockarie, Akosua Bema Kumah, Alex Bismark Atta-Owusu, Mordecai Eshun, Bernice Akua Frimpong, Beatrice Bachella, George Nkrumah Osei

**Affiliations:** a Department of Medical Laboratory Science, School of Allied Health Sciences, University of Cape Coast, Cape Coast, Ghana; b Department of Internal medicine and Therapeutics, School of Medical Sciences, University of Cape Coast, Cape Coast, Ghana

**Keywords:** Sleep quality, Pittsburgh sleep quality index, Students, Age, Ghana, Sex

## Abstract

**Introduction:**

Sleep plays a crucial role in health, well-being, and academic performance. Despite the recognized importance of good sleep for students, there is a need for a deeper understanding of the sleep problems faced by university students to inform effective campus support services and interventions. This study aimed to evaluate sleep quality among university students by assessing differences in key sleep parameters between sex and age groups.

**Methodology:**

A cross-sectional study was conducted to assess sleep quality using the validated Pittsburgh Sleep Quality Index among 295 undergraduate students at the University of Cape Coast, Ghana. Demographic characteristics of age and sex were also collected. Data were analyzed using descriptive statistics, Pearson's correlation, and cross-tabulations to compare sleep quality scores between sex and age groups.

**Results:**

The study included 295 undergraduate students aged 20–29 years, of which 53.2 % were male. Overall, 48.5 % of participants exhibited poor sleep quality (PSQI >5). The distribution of global PSQI scores varied by age group, with those aged 25, 27, 28, and 29 reporting higher rates of poor sleep quality. Mean scores on global PSQI scores and other components, including sleep duration and habitual sleep efficiency, were highest among those aged 25 years. Female students were more likely to experience poor sleep quality than male students (51.4 % vs. 45.2 %). Key sleep parameters such as sleep latency, disturbances, and daytime dysfunction showed significant associations with increasing age, although the correlations were weak. Global PSQI scores significantly correlated with both age (r = 0.378, p = 0.001) and sex (r = 0.212, p = 0.001).

**Conclusion:**

This study revealed a high prevalence of poor sleep quality among the respondent, with sleep disturbances and duration disproportionately affecting their sleep quality. These findings underscore the need for universities to prioritize promoting healthy sleep habits and addressing diverse sleep component issues within their student populations.

## Background

University students often grapple with mental health challenges, high stress levels, and subpar academic performance, often attributed to a combination of psychological difficulties and poor sleep quality (Arbabisarjou et al., 2016, Gaultney, 2010, Gobin et al., 2015, Haregu et al., 2015, James et al., 2011, Nadeem et al., 2024, Rose et al., 2015, Yang et al., 2003, Yang and Smallfield, 2020, Zochil, 2013, Armand et al., 2021, Zochil and Thorsteinsson, 2020). Poor sleep quality is characterized by the inability to initiate, maintain, or achieve restful sleep (Atalay, 2011). Key indicators of decreased sleep quality include reduced sleep duration, morning fatigue, and difficulties with sleep initiation and maintenance, which collectively contribute to disrupted sleep continuity (Buysse et al., 1989, Wang, 2021, Kline, 2013). Research has underscored the critical role of sleep in numerous physiological and cognitive processes, including immune function, metabolism, memory, learning, mood, behavior, and performance (Doyle and Zakrajsek, 2013, Džaferović and Ulen, 2018, James et al., 2011, Nadeem et al., 2024, Ramar et al., 2021, Yang and Smallfield, 2020) This highlights the significance of sleep for overall health and well-being.

Research indicates that 16–23 % of young adults generally experience poor sleep quality (Taylor et al., 2011). However, the prevalence of poor sleep quality is significantly higher among university students compared to other young people (Brown et al., 2002, Cheng et al., 2012). Alarmingly, studies have reported even greater rates of poor sleep quality in specific student subgroups, such as 75.8 % among medical students (Džaferović and Ulen, 2018), 64.7 % among medical students (Khan Awan et al., 2018), and 60 % in a broader university student sample (Lund et al., 2010). These elevated rates suggest that university students face an elevated risk of experiencing poor sleep quality compared to their peers.

University students frequently report experiencing sleep-related issues, with studies identifying daytime sleepiness as a major contributor (Cates et al., 2015, Khan Awan et al., 2018, Surani et al., 2015, Yang and Smallfield, 2020). Research has also shown a significant association between poor sleep quality and various sleep disturbances in this population (Surani et al., 2015). The consequences of disrupted sleep among university students can be severe, as poor sleep duration has been linked to poorer mental health, lower academic performance, increased risk of academic failure, and compromised learning abilities (Batten et al., 2020, Hershner and Chervin, 2014)This is because sleep deprivation can negatively impact the key learning-related brain processes of acquisition, consolidation, and recall (Cates et al., 2015), posing a significant challenge for university students.

These concerning findings underscore the critical need to better understand the nature and prevalence of sleep problems experienced by university students. This study assessed sleep quality across sex and age groups within the university student population. The findings will provide valuable insights into the prevalence and nature of sleep problems faced by university students, informing the development of targeted interventions to promote optimal sleep health in diverse demographic profiles.

## Methodology

### Study design, site, population, and sample size

A cross-sectional study design was employed to gather information on sleep quality among regular undergraduate students at the University of Cape Coast, a large public university located in the Central Region of Ghana. The study was conducted from March 2022 to June 2022.

The sample size was calculated based on an estimated 25 % prevalence of poor sleep quality, with a 5 % margin of error and a confidence level of 95 % and using the formula, [Z^2^p (1-p)]/C^2^. An estimated sample size of 289 was obtained and this was rounded up to 300 to compensate for participant non-response. A convenience sampling technique was used to select a total of 295 participants aged 20–29 years. The study included regular undergraduates from the University of Cape Coast who were able and willing to participate by completing the necessary study questionnaire. However, students who were already receiving treatment for sleep-related disorders and/or other medical issues that could substantially affect their sleep patterns were excluded from the study. Additionally, non-consenting students were excluded.

### Ethical considerations

The study protocol was reviewed and approved by Cape Coast Teaching Hospital Ethical Review Committee (ethical clearance ID: CCTHERC/EC/2021/020). All methods were carried out in accordance with relevant guidelines and regulations. Informed consent was obtained from all participants and confidentiality was also observed throughout the entire study.

### Sampling and data collection procedure

A structured approach was followed to gather the necessary data for the study. First, all eligible students were fully informed about the objectives of the study, the procedures participants would be asked to follow, and the potential benefits and rights of the participants. Informed consent was then obtained from participants. The participants were asked to provide their demographic information (age and sex) after consenting to participate in the study. Finally, the participants were administered the Pittsburgh Sleep Questionnaire Index (PSQI), a well-validated self-report instrument used to comprehensively assess various aspects of sleep quality, to complete.

### Assessment of sleep quality using the Pittsburgh Sleep Questionnaire Index (PSQI)

The PSQI is a widely used, reliable, and well-validated self-report questionnaire that evaluates seven key components of sleep quality: subjective sleep quality, sleep latency, sleep duration, sleep efficiency, sleep disturbances, sleep medication, and daytime dysfunction and each component is scored on a 0–3 scale (Buysse et al., 1989). The PSQI offers a reliable and standardized approach for distinguishing "good" sleep from "poor" sleep using a simple global scoring index (Pilz et al., 2018). The global PSQI score, calculated by adding the seven component scores, ranges from 0 to 21. Scores greater than 5 indicate poorer sleep quality. The English version of the index was used in this study.

### Data analysis

Data was first entered into Microsoft Excel and analyzed using Statistical Package for Social Sciences (SPSS version16 software). Crosstabulation was used to present the distribution of result based on age group as against global PSQI score ≤ 5 and that > 5. The global PSQI score, the scores for the seven domains of sleep, sex and age were compared using means and standard deviation. To determine the association between age and the 7 component that constitute global PSQI, Pearson correlation coefficient was performed. A p-value of less than 0.05 was considered statistically significant. All analyses were done at a 95 % confidence interval.

## Results

### **The distribution of age and sex in relation to Global PSQI scores among study participants**

The study involved 295 participants, with the majority being male (n = 157, 53.2 %). The minimum and maximum global PSQI scores were 1 and 17, respectively (Table 1). Of the total participants, 143 (48.5 %) reported experiencing difficulties in some aspect of their sleep (PSQI > 5).

**Table 1:** Crosstabulation showing the distribution of age and sex against global PSQI score.

Characteristics	Global PSQI score (Min-1, Max-17)		Total
	≤ 5	> 5	
Age (years)			
20	12	3	**15**
21	14	12	**26**
22	14	10	**24**
23	19	5	**24**
24	19	15	**34**
25	10	18	**28**
26	13	12	**25**
27	20	23	**43**
28	22	30	**52**
29	9	15	**24**
Total	**152**	**143**	**295**

Sex			
Male	85	72	**157**
Female	67	71	**138**
Total	**152**	**143**	**295**

An analysis of global PSQI scores across age groups revealed that for ages 25, 27, 28, and 29, the majority of respondents reported sleep difficulties (Table 1). However, for age group 26, the number of participants with sleep difficulties (12) was nearly identical to the number of participants without sleep difficulties (13).

Among the 157 male participants, the majority (n = 85, 54.1 %) reported no sleep difficulties (PSQI score ≤ 5). In contrast, the majority of female respondents (n = 71, 51.4 %) experienced sleep difficulties (Table 1).

### Pittsburgh Sleep Quality Index (PSQI) global and component score profiles across various age groups

From age 20–25, the mean global PSQI score correspondingly increased from 1.60 ± 0.51–7.71 ± 3.96(Table 2). On average, participants aged 20–22 reported minimal or no sleep difficulties (PSQI score ≤ 5) in all areas. By analyzing the individual PSQI component scores, which range from 0 (no difficulty) to 3 (severe difficulty), we identified sleep latency, sleep duration, and sleep disturbances as areas of concern across the age groups (Table 2).

**Table 2:** The means and standard deviation of the seven (7) components of PSQI among the various age group.

Age	G PSQI Score	C1	C2	C3	C4	C5	C6	C7
	Mean ± SD	Mean ± SD	Mean ± SD	Mean ± SD	Mean ± SD	Mean ± SD	Mean ± SD	Mean ± SD
20 (n = 15)	1.60 ± 0.51	0.73 ± 0.88	0.47 ± 0.64	0.87 ± 0.35	0.73 ± 1.03	1.07 ± 0.46	0.13 ± 0.35	0.40 ± 0.51
21 (n = 26)	3.19 ± 0.57	0.81 ± 0.63	0.81 ± 0.85	1.15 ± 0.83	1.12 ± 1.40	1.00 ± 0.63	0.15 ± 0.61	0.73 ± 0.83
22 (n = 24)	4.71 ± 0.46	1.04 ± 0.75	0.96 ± 0.91	0.83 ± 0.70	0.63 ± 1.06	1.25 ± 0.53	0.08 ± 0.28	0.62 ± 0.58
23 (n = 24)	5.67 ± 0.48	0.79 ± 0.72	0.75 ± 0.85	1.13 ± 0.74	0.58 ± 0.88	1.04 ± 0.46	0.08 ± 0.28	0.46 ± 0.66
24 (n = 34)	7.24 ± 0.70	0.91 ± 0.75	0.74 ± 0.83	1.06 ± 0.78	0.97 ± 1.19	1.09 ± 0.45	0.24 ± 0.65	0.68 ± 0.84
25 (n = 28)	7.71 ± 3.96	0.93 ± 0.60	0.82 ± 0.82	1.29 ± 0.98	1.14 ± 1.27	1.25 ± 0.52	0.18 ± 0.61	0.96 ± 0.79
26 (n = 25)	6.24 ± 2.57	0.92 ± 0.81	1.20 ± 0.82	1.04 ± 0.84	0.44 ± 0.92	1.24 ± 0.52	0.36 ± 0.81	0.68 ± 0.63
27 (n = 43)	5.70 ± 2.87	0.93 ± 0.70	1.12 ± 0.82	1.19 ± 0.88	0.95 ± 1.25	1.35 ± 0.53	0.16 ± 0.53	0.77 ± 0.61
28 (n = 52)	6.85 ± 3.29	0.94 ± 0.67	1.10 ± 0.96	0.90 ± 0.72	0.65 ± 0.99	1.42 ± 0.57	0.27 ± 0.72	0.88 ± 0.65
29 (n = 24)	6.58 ± 2.43	0.92 ± 0.28	1.17 ± 0.70	1.17 ± 0.64	0.75 ± 1.19	1.33 ± 0.48	0.21 ± 0.42	0.63 ± 0.50

C1-Subjective sleep quality, C2-sleep latency, C3-sleep duration, C4-habitual sleep efficiency, C5-sleep disturbances, C6-use of sleeping medication, and C7-daytime dysfunction

**From age 26–29, the mean score for sleep latency consistently remained above 1.** Similarly, except for ages 22 and 28, the mean score for sleep duration also remained greater than 1. Moreover, the mean score for sleep disturbances was consistently 1 or higher for all age groups. The age 25 group recorded the highest mean global PSQI score (7.71 ± 3.96), with sleep duration (1.29 ± 0.98), habitual sleep efficiency (1.14 ± 1.27), and sleep disturbances (1.25 ± 0.52) contributing significantly to this observation (Table 2).

### Pattern of sleep quality or difficulty among the sexes

On average, the mean global PSQI score for both males (5.31 ± 2.56) and females (6.51 ± 3.01) exceeded the cutoff point of 5, indicating that both sexes experienced sleep difficulties in some areas (Table 3).

**Table 3:** The mean and standard deviation of the seven (7) components of PSQI among sexes.

Sex	C1	C2	C3	C4	C5	C6	C7	PSQI
	Mean ± SD	Mean ± SD	Mean ± SD	Mean ± SD	Mean ± SD	Mean ± SD	Mean ± SD	Mean ± SD
Male (n = 157)	0.87 ± 0.61	0.98 ± 0.90	1.15 ± 0.79	0.83 ± 1.15	1.19 ± 0.50	0.16 ± 0.53	0.67 ± 0.66	5.31 ± 2.56
Female (n = 138)	0.94 ± 0.76	0.91 ± 0.80	0.96 ± 0.77	0.78 ± 1.13	1.28 ± 0.58	0.24 ± 0.63	0.78 ± 0.71	6.51 ± 3.01
Total (n = 295)	0.91 ± 0.68	0.95 ± 0.85	1.06 ± 0.78	0.81 ± 1.14	1.23 ± 0.54	0.20 ± 0.58	0.72 ± 0.68	5.87 ± 5.87

C1-Subjective sleep quality, C2-sleep latency, C3-sleep duration, C4-habitual sleep efficiency, C5-sleep disturbances, C6-use of sleeping medication, and C7-daytime dysfunction

Specific areas with higher mean score values for males included sleep duration (1.15 ± 0.79) and sleep disturbances (1.19 ± 0.50). However, among female participants, only sleep disturbances showed a higher mean score (1.28 ± 0.58) (Table 2)

### Relationship between age and the seven components of sleep that constitute the global PSQI

A statistically significant association was found between age and specific sleep parameters, such as sleep latency, sleep disturbances, and daytime dysfunction. However, the relationship between these variables and age exhibited a weak degree of correlation (Table 4).

**Table 4:** Correlation between age group and the seven (7) components of PSQI.

The 7 components of PSQI	Age	
	r	p-value
Subjective sleep quality	0.051	0.384
Sleep latency	0.192	0.001
Sleep duration	0.022	0.701
habitual sleep efficiency	−0.038	0.521
Sleep disturbances	0.230	0.000
Use of sleeping medication	0.076	0.195
Daytime dysfunction	0.116	0.047
	
Demographic characteristics	**Global PSQI Score**	
	**r**	**p-value**
Age	0.378	0.001
Sex	0.212	0.001

Additionally, the global PSQI scores demonstrated a significant moderate correlation with both age (r = 0.378, p = 0.001) and sex (r = 0.212, p = 0.001) (Table 4).

## Discussion

Sleep quality is essential for clinical education, as it directly affects cognitive function, academic performance, emotional regulation, and overall health among students (Batten et al., 2020, Nadeem et al., 2024). This study examined sleep quality among 295 undergraduate students using the Pittsburgh Sleep Quality Index (PSQI).

Overall, nearly half (48.5 %) of participants reported experiencing some degree of sleep difficulty, indicating a high prevalence of poor sleep quality (PSQI > 5). This prevalence was higher than that observed in similar studies among occupational therapy (10.5 %) and medical students (39.5 % and 19.2 %) (Feng et al., 2005, Surani et al., 2015, Yang and Smallfield, 2020), suggesting an increased risk of poor sleep quality among this population compared to other university students. This may be attributed to the increasingly rigorous academic demands placed on students as they progress through their university programs. However, the observed prevalence of poor sleep quality was lower than the 75.8 % and 64.7 %, rates reported by Džaferović & Ulen and Awan et al. among medical students (Džaferović and Ulen, 2018, Khan Awan et al., 2018) and the 60% found by Lund et al. among university students (Lund et al., 2010). These findings suggest that sleep issues disproportionately burden certain student populations, and patterns may vary depending on the contextual and demographic characteristics of each study population.

The study found that individuals between the ages of 20–24 and 26 primarily reported an absence of sleep difficulties. In contrast, respondents in the 25, 27, 28, and 29 age groups mainly reported experiencing sleep issues, suggesting that sleep problems may become more prevalent among university students as they progress in their academic careers.

Specific sleep component analysis revealed that daytime dysfunction and the use of sleep medication were generally non-issues across age groups, with mean scores less than 1. This suggests that daytime dysfunction and reliance on sleep aids do not appear to be major contributors to reported sleep problems among this student population, according to their self-assessments. This finding differs from previous research that identified daytime sleepiness as a common issue impacting academic performance and participation (Cates et al., 2015, Khan Awan et al., 2018, Surani et al., 2015, Yang and Smallfield, 2020). The disparity between results may be attributed to variations in sleep patterns among student populations due to differing academic loads and environments.

However, consistently elevated scores for sleep disturbances (≥1) across all ages highlight sleep disturbances as a widespread concern negatively impacting university students. This finding aligns with Surani et al.'s study, which also observed a significant association between poor sleep quality and sleep disturbances (Surani et al., 2015). Additionally, elevated sleep duration scores (≥1) were observed from ages 21–29, except for ages 22 and 28, further corroborating sleep duration as another major sleep issue facing university students. Disrupted sleep duration has been previously linked to poorer mental health, lower grade point averages, increased risk of academic failure, and compromised learning at the university level (Batten et al., 2020, Hershner and Chervin, 2014). Notably, sleep disturbances and duration were the primary contributors to the highest global PSQI score in the 25-year age group, adding to growing evidence that the mid-twenties can represent a particularly vulnerable period for sleep problems among undergraduate university students.

The results of this study indicated that both male and female university students experienced poor sleep quality on average, with global PSQI scores exceeding the clinical cutoff of 5. This was similarly observed by Cates et al. among pharmacy students (Cates et al., 2015), signifying that poor sleep is a widespread concern impacting the student population regardless of gender. However, a higher mean global score for females coupled with a higher incidence of poor sleep quality observed among females (51.4 %) compared to males suggests that females may be at increased risk for more severe or frequent sleep difficulties. These findings resonate with research by Cates et al., Surani et al., and Gaultney, which also observed poorer sleep quality among female than male students, demonstrating that sleep problems may disproportionately impact female populations in the university context (Cates et al., 2015, Gaultney, 2010, Surani et al., 2015). Notably, sleep disturbances were a shared primary complaint across genders, with higher mean scores observed among females. This suggests that interventional approaches targeting triggers of sleep disturbances, such as stress, anxiety, and mental wellness, may provide benefits regardless of sex or other demographic characteristics.

## Conclusion

The study revealed a higher prevalence of poor sleep quality among regular undergraduate students in a Ghanaian tertiary institution, with sleep disturbances and duration significantly impacting their sleep quality. Female university students may be at increased risk for more severe or frequent sleep difficulties compared to male students. Sleep disturbances were found to disproportionately impact female undergraduates, suggesting that interventions targeting triggers of sleep disturbances, such as stress, anxiety, and mental wellness, may be particularly beneficial for this population.

These findings underscore the importance of fostering healthy sleep habits and considering various components of sleep quality to optimize the well-being and academic success of university students.

### Strengths, limitations and recommendations

The strengths of this study lie in its comprehensive assessment of sleep quality among university students. The researchers used the well-validated Pittsburgh Sleep Quality Index (PSQI) to thoroughly evaluate various aspects of sleep, including sleep latency, duration, and disturbances. Additionally, the study considered demographic factors such as gender and age, providing valuable insights into the specific sleep patterns and challenges faced by different groups within the student population.

However, the study also had some limitations. The research was conducted at a single university, which may limit the generalizability of the findings to the broader population of students in Ghana or other regions. Furthermore, the cross-sectional design of the study only provided a snapshot of sleep quality at a particular time, and could not establish causal relationships or track changes in sleep patterns over time.

To address these limitations, the researchers suggest expanding the study setting to include multiple universities or tertiary institutions in Ghana. This would enhance the generalizability of the findings and provide a more comprehensive understanding of sleep quality among university students. Additionally, the researchers recommend implementing a longitudinal study design, which would aid in investigating changes in sleep quality over time and identify potential causal factors.

## Ethical consideration

Ethical approval was obtained from University of Cape Coast Institutional Review Board**.** All methods were carried out in accordance with relevant guidelines and regulations. Informed consent was obtained from all participants and confidentiality was also observed throughout the entire study.

## Funding

The authors received no specific funding for this work.

## CRediT authorship contribution statement

**Beatrice Bachella:** Writing – review & editing, Methodology, Data curation, Conceptualization. **George Nkrumah Osei:** Writing – review & editing, Writing – original draft, Visualization, Methodology, Conceptualization. **Akosua Bema Kumah:** Writing – review & editing, Methodology, Data curation, Conceptualization. **Alex Bismark Atta-Owusu:** Writing – review & editing, Methodology, Data curation, Conceptualization. **Mordecai Eshun:** Writing – review & editing, Methodology, Data curation, Conceptualization. **Bernice Akua Frimpong:** Writing – review & editing, Methodology, Conceptualization. **David Larbi Simpong:** Writing – review & editing, Writing – original draft, Visualization, Validation, Supervision, Project administration, Methodology, Investigation, Formal analysis, Conceptualization. **Ansumana Bockarie:** Writing – review & editing, Writing – original draft, Methodology, Conceptualization.

## Conflicts of interest

Authors declare no conflicting interest.

## Data Availability

All data generated or analyzed during this study are available upon request from the corresponding author.
